# Assessment of interprofessional obstetric and midwifery care from the midwives’ perspective using the Interprofessional Collaboration Scale (ICS)

**DOI:** 10.3389/fpsyg.2023.1143110

**Published:** 2023-05-22

**Authors:** Anja Alexandra Schulz, Markus Antonius Wirtz

**Affiliations:** Research Methods in the Health Sciences, University of Education Freiburg, Freiburg, Germany

**Keywords:** interprofessional collaboration, midwifery care, woman-centered care, psychometric evaluation, confirmatory factor analysis

## Abstract

**Introduction:**

Interprofessional collaboration of physicians and midwives is essential for appropriate and safe care of pregnant and parturient women as well as their newborns. The complexity of woman-centered care settings requires the continuous exchange of information and the coordinated implementation of multi-and interprofessional care concepts. To analyze the midwives’ perspective on the multi-and interprofessional care process during pregnancy, birth and postpartum period, we aimed to adapt and psychometrically evaluate the Interprofessional Collaboration Scale (ICS).

**Methods:**

The ICS (13 items) was answered by 299 midwives for (i) prenatal and postpartum care as well as (ii) perinatal care. Three items on equitable communication (EC) identified in qualitative interviews with *N* = 6 midwives were added as further aspects of quality in collaborative midwifery care. Confirmatory factor analysis was used to test competing theoretically hypothesized factorial model structures, including both care settings simultaneously, i.e., birth and prenatal/postpartum.

**Results:**

A two-dimensional structure assuming the 13 original ICS items and the 3 items on EC as psychometric distinct item groups accounts for the data best. After deleting 5 ICS items with insufficient indicator reliability, a very good-fitting model structure was obtained for both prenatal/postpartum as well as perinatal care: *χ^2^*_df = 192_ = 226.35, *p* = 0.045, CFI = 0.991, RMSEA = 0.025 (90%CI: [0.004; 0.037]). Both the reduced ICS-R and the EC scale (standardized response mean = 0.579/1.401) indicate significantly higher interprofessional collaboration in the birth setting. Responsibility in consulting, attitudes toward obstetric care and frequency of collaboration with other professional groups proved to be associated with the ICS-R and EC scale as expected.

**Discussion:**

For the adapted ICS-R and the EC scale a good construct validity could be confirmed. Thus, the scales can be recommended as a promising assessment for recording the collaboration of midwives with physicians working in obstetric care from the perspective of midwives. The instrument provides a validated assessment basis in midwifery and obstetric care to identify potentially divergent perspectives within interprofessional care teams in woman’s centered care.

## 1. Introduction

The care of pregnant women, women in labor, women who have recently given birth, and newborns takes place in a multidisciplinary care context (International Confederation of Midwives, 2014; Hansson et al., 2019). All professions involved in maternal and child care share the common goal of providing high-quality, safe, and efficient health care (Angelini et al., 2012; Tunçalp et al., 2015). Additionally, health care professionals in (non-)clinical obstetrics face the challenge of fulfilling the demands of modern obstetrics and the increasingly complex care processes with sometimes limited care capacity and maximally utilized (human) resources (Shamian, 2014; WHO, 2016).

The purposeful linking of profession-specific knowledge and skills in the sense of integrated care practice or interprofessional care may contribute to ensure the required status quo of quality of care (Shamian, 2014; WHO Regional Office for Europe, 2015; Freytsis et al., 2017). Interprofessional collaboration (IC) of midwives and physicians is defined as “a process in which midwives and physicians work together toward a common purpose: to provide safe, effective, patient-centered care for women and their families, guided by shared rules and structures, both formal and informal, which govern a mutually beneficial relationship, a relationship which seeks to optimize the context in which the collaboration is convened” (Smith, 2015). Successful IC supports the development of a common understanding in terms of a continuum of care in which competing or conflicting ways of working are avoided (McFarland et al., 2020; Stahl and Agricola, 2021). Insufficient cooperation within the obstetric staff is be perceived by mothers as a negative experience during the care process (Cornthwaite et al., 2013).

### 1.1. Midwives’ and physicians’ perspective on interprofessional collaboration

Professional groups involved in obstetric care generally consider that the benefits of IC predominate (Aquino et al., 2016). Both midwives and physicians perceive a positive effect in the case of successful IC with regard to woman-centered care outcomes (Cornthwaite et al., 2013; Aquino et al., 2016). The professional group affiliation is of particular importance when assessing the individual evaluation of IC. Especially, in the clinical setting physicians’ perceptions of IC in everyday care with midwives and nurses proved to be more positive than vice versa (Warmelink et al., 2017; Romijn et al., 2018). In contrast, non-medical health care professionals generally have a more positive attitude toward IC than physician staff (Sollami et al., 2015).

Because professions understand the IC differently, the practice of IC is perceived differently by these and the respective expectations may differ (Lingard et al., 2012; Sollami et al., 2015). Accordingly, endpoints of the assessment must be defined and operationalized clearly and unambiguously (IC attitude or IC perception) to allow for a valid comparison between professional groups (Lingard et al., 2012; Sollami et al., 2015).

Challenges in implementing IC in the clinical obstetric care setting are well documented and stringently reported regardless of professional group perspective. In general, pronounced hierarchical structures, fragmentation of care, lack of respect and trust, and unclear areas of responsibility and authority are key barriers to implement IC (Smith, 2015; Aquino et al., 2016). Midwives perceive their work environment as tense with a high risk of conflict (McFarland et al., 2020). Professional dissonance, caused by discrepancies in professional ethics or expectations of, e.g., communication structures and coordination mechanisms, is considered a central cause (Smith, 2015; Hansson et al., 2022). The overall heterogeneous professional basic understanding (physiological vs. pathological) as well as competing birth concepts (home birth vs. clinical birth) and traditionally determined concepts of care (trust in the normality of birth vs. birth as a high-risk event) between midwives and physicians may also be influential. These aspects may enhance feelings of demarcation between professional groups and impede a shared vision or philosophy of care (Reiger, 2008; Reiger and Lane, 2009; Behruzi et al., 2017; McFarland et al., 2020).

In addition to the demanding and complex care setting, high fluctuation, inadequate professional resources, and poor work climate, conflicting ideologies within the team and role conflicts may additionally negatively influence the experience of emotional demands, increase job-related stress, and negatively affect job satisfaction (Hunter, 2004; Nedvědová et al., 2017; Bloxsome et al., 2019). Fostering IC also improves the organizational and psychosocial work environment of health professionals and is positively associated with job satisfaction (Weller et al., 2014; Dinius et al., 2020).

### 1.2. Challenges in analyzing effects of interprofessional collaboration in woman-centered maternity and obstetric care

The effectiveness of working in collaborative care teams in obstetrics in terms of woman-related healthcare outcomes proved to be limited (Homer et al., 2001; Sandall et al., 2016; Lee et al., 2021). Care within an interprofessional continuity of care model (midwife-led continuity model) is associated with a reduction in (i) instrumental vaginal births (mean RR = 0.90; 95% CI: [0.83; 0.97]), (ii) local analgesia (mean RR = 0.85; 95% CI: [0.78; 0.92]), (iii) preterm birth (mean RR = 0.76; 95%- CI: [0.64; 0.91]), and (iv) miscarriage before and after 24 weeks of gestation (mean RR = 0.84; 95% CI: [0.71; 0.99]) (Sandall et al., 2016). In addition, the likelihood of spontaneous natural delivery is increased (mean RR = 1.05; 95%CI: [1.03; 1.07]) (Sandall et al., 2016). However, some research results also indicate negative effects of IC, i.a. reducing productivity or enhancing restricted decision-making processes due to the necessity of more complex coordination processes (Mitchell et al., 2011; Kaba et al., 2016).

When considering the reported effects, it is important to take into account that inconsistent foundations for the operationalization of IC make the interpretation and comparability of the effects difficult (Reeves et al., 2011; Langer et al., 2012; Kaba et al., 2016; Reeves et al., 2017). The types and practices of IC vary widely from (i) simple information through (ii) enabling and generating synergies of the professions involved to (iii) joint decision-making and action processes (Gerber et al., 2018). Furthermore, the construct IC is often analyzed as a sub-aspect of a multimodal intervention (e.g., integrated care) or as a facet within the scope of action of occupational psychological processes (Stahl et al., 2019). This fact, combined with the paucity of study results based on experimental studies, makes it difficult to classify the impact of IC in terms of patient-relevant outcomes, patient safety, efficiency, and improved quality of care in general (Mitchell et al., 2011; Kaba et al., 2016; Reeves et al., 2017).

### 1.3. Operationalization of interprofessional collaboration of midwives and physicians

IC in the health care sector is primarily assessed using self-rating instruments (Walters et al., 2016). The focus is predominantly on capturing IC between physicians and nurses in different health care settings (Sollami et al., 2015). Most instruments assess attitudes related to IC [e.g., Jefferson Scale of Attitude towards Physician-Nurse Collaboration (JSAPNC) (Hojat et al., 1997)], while a smaller number operationalize perceived IC in interprofessional teams [e.g., Nurse-Physician Collaboration Scale (NPCS) (Ushiro, 2009), Collaboration Practice Scale (CPS) (Weiss and Davis, 1985), Collaboration and Satisfaction About Care Decision Scale (CSACDS) (Baggs, 1994)].

The instruments assess not only the frequency of conferences with other professions, but also sub-facets of collaborative relationship, the organizational climate or information management processes (Ushiro, 2009). Validation steps with samples from allied health staff (e.g., midwives) are missing (Peltonen et al., 2020). Furthermore, a limited examination of psychometric properties of assessment instruments is to be acknowledged (Peltonen et al., 2020).

The Interprofessional Collaboration Scale (ICS) takes a generic approach to capture IC between different health care professions (Kenaszchuk et al., 2010). The multiple-group assessment was developed primarily for three professions in clinical settings: physicians, nurses, and other regulated health care professionals (e.g., speech therapists, dietitians, physical therapists). In successive validation steps, the three-factorial structure of the questionnaire: (i) *Communication*, (ii) *Accommodation*, (iii) *Isolation*, was confirmed also for the German version (Vittadello et al., 2018). However, shortcomings in model fit were found for the group of allied health personnel (occupational and physical therapists, pharmacists, social workers). The authors recommend psychometric testing not for the allied health personnel in general. Instead, the analysis should be specific for each occupational group that belong to the more general population of health care workers (Kenaszchuk et al., 2010). Because of the generic developmental approach, the ICS can be considered a relevant operationalization approach for assessing IC in obstetric care between midwives and physicians.

### 1.4. Properties of the German midwifery care system

The unique properties of the German midwifery system should be taken into account when investigating IC in the midwifery and obstetric care setting. All insured women in Germany have a statutory entitlement to midwifery care during pregnancy, childbirth, the postpartum period, and during breastfeeding. This includes activities such as preventive examinations, help with pregnancy complaints, care of sutures and birth injuries, postpartum care, and conducting newborn screening. In addition, midwives are responsible for the independent management of physiological births without risk (§ 1 Midwives Law). Furthermore, there are different work structures, whereby midwives work as employees (mainly clinical obstetrics), freelancers (e.g., out-of-hospital obstetrics, prenatal care, retraining) or both. Thus, a variety of midwifery activities are provided in different care settings (prenatal, perinatal, postpartum) in multi-and interdisciplinary care teams (specialists in obstetrics and gynecology, pediatricians, midwives). A differentiated assessment and comparison of midwives’ perspectives on IC with physicians in clinical and out-of-hospital care of pregnant women, mothers, and women in childbirth has not yet been conducted (O’Reilly et al., 2017).

### 1.5. Study aims and research questions

To assess IC of physicians and midwives in clinical and out-of-hospital care settings in Germany, we adapted the existing German version of the ICS (Vittadello et al., 2018) to the context of midwifery care considering further aspects to ensure content-validity. The analysis was divided in two steps: First, psychometric evaluation of the scale properties of the adapted ICS supplemented by additional items on equitable communication (EC) between midwives and physicians; Secondly, evaluation of the IC from the perspective of midwives in clinical and out-of-hospital care settings on scale and item level. The bivariate relationship with other IC-associated characteristics was analyzed exploratorily. Thus, the following research questions were investigated:

1. Are the responses on the 13 ICS items and the 3 EC items determined by a four-factor structure 4-DIM model (accommodation, isolation, communication, equitable communication)?
2. Do midwives’ views of IC with physicians differ between care settings (prenatal/postpartum vs. perinatal) on item and scale level?
3. Are the ICS scores associated with

- midwives’ job satisfaction?
- perceptions and attitudes toward the obstetric care process and professional responsibilities?
- the frequency of collaboration with other professional groups?

## 2. Materials and methods

The present study is a follow-up study of the research project “Structural analysis of midwifery care in the rural district of Ortenau (Southwest Germany)” which was approved to be ethically appropriate by the Ethics Committee of the German Psychological Society (DGPs; Ref: MAW 022019). The study was conducted from April to May 2020 as a cross-sectional online survey using the SoSci Survey tool (anonymous online questionnaire). No personal data were collected. Only characteristics of the individual work situation (scope and duration of work, field of activity, federal state) were recorded. Accordingly, the local ethics committee did not require a separate ethics vote for this study arm. All participating midwives were fully informed about study conditions (especially data privacy and protection) and participant rights. Confirmation of informed consent was obtained prior to completion of the questionnaire.

### 2.1. Sample

Midwives were recruited in a two-stage selection process. Ad hoc samples of independent and employed midwives in clinical and non-clinical care were drawn in all 16 federal states of Germany (primary sampling units). In addition, recruitment was supported by multipliers at the level of regional and national associations.

*N* = 468 midwives could be enrolled. Of these, *N* = 325 (69.4%) completed the online questionnaire. Twenty-six of these cases had to be excluded from the sample due to premature termination of questionnaire processing. Accordingly, *N* = 299 (63.9%) were included in the final data analysis. The questionnaires were completely answered except for single missing data (maximum of missing data on the scale items *N* = 8 or 0.4%).

### 2.2. Instruments

The ICS (Kenaszchuk et al., 2010) is a self-report tool that was developed to assess core aspects of IC between two or more professional groups in health care (e.g., nurses, doctors, allied health professionals). Each of the 13 scale items (Table 1) is answered on a 4-point rating scales ranging from “1” – “strongly disagree” to “4” – “strongly agree”. Factor analysis revealed a three-factor structure of the self-report tool: perceptions of *Communication*, *Isolation*, and *Accommodation* proved to be distinguishable. Nevertheless, the three identified factors were highly correlated (e.g.: nurses rating collaboration with physicians: *r* = 0.75–0.86). Composite reliability proved to be acceptable for *Communication* and *Isolation* (*ρ*_c_ = 0.76 in each case), and good for *Accommodation* (*ρ*_c_ = 0.85). In the present study, according to the basic conception of the instrument, the professional groups *physicians* and *midwives* were placed in the item templates. The assessment of IC of these two professional groups in the care of pregnant and childbearing women was made from the perspective of midwives. The content validity of the ICS for IC in prenatal and obstetric woman-centered care could be substantiated by preceding qualitative interviews with *N* = 6 midwives. The content of each item corresponded with statements made by the midwives interviewed. However, in the interviews, midwives placed emphasis on the importance of equitable interprofessional communication and team spirit. In order to take these aspects into account, three additional items were formulated which were intended to ensure the completeness of the content spectrum of IC in obstetrics (Table 1; EC-01 to EC-03). These items were answered on 6-point Likert scales. According to the response range of the ICS items, response categories were coded from “1” – “strongly disagree” to “4” – “strongly agree” (intermediate levels: “1.6” – “mostly disagree”, “2.20” – “rather disagree”, “2.80” – “rather agree”, “3.40” – “mostly agree”).

**Table 1:** Mean values and stability of the items of the original ICS and the EC scale in prenatal and postpartum care (PPC) as well as in birth (BC) care in the total sample of *N* = 299 midwives.

	M (PPC)	SD (PPC)	M (BC)	SD (BC)	r_PPC, BC_^2^	SD (DIF)^4^	SRM^5^	r_it_^6^ (PPC | BC)	α (PPC | BC)
*Interprofessional collaboration scale-R*	*2.25*	*0.687*	*2.55*	*0.588*	*0.668* ^***^	*0.527*	*0.579* ^***^		*0.920 | 0.874*
ICS-01: Midwives have a good understanding with physicians about our respective responsibilities	2.51	0.880	2.90	0.723	0.336^***^	0.933	0.420^***^		
ICS-02: Physicians are usually willing to take into account the convenience of midwives when planning their work	2.15	0.810	2.63	0.802	0.311^***^	0.946	0.513^***^	0.713 | 0.546	
ICS-03: I feel that woman and newborn care are adequately discussed between midwives and physicians^1^	2.33	0.864	2.62	0.840	0.424^***^	0.915	0.315^***^	0.744 | 0.681	
ICS-04: The physicians and midwives have similar ideas about how women and newborn should be treated	2.40	0.815	2.51	0.813	0.492^***^	0.820	(0.130^*^)^3^	0.651 | 0.641	
ICS-05: Physicians are willing to discuss midwives’ issues	2.34	0.903	2.65	0.836	0.560^***^	0.818	0.372^***^	0.799| 0.695	
ICS-06: Physicians cooperate with the way we organize midwifery	2.40	0.835	2.71	0.726	0.418^***^	0.848	0.367^***^	0.755 | 0.647	
ICS-07: Physicians would be willing to cooperate with midwifery practices	2.15	0.782	2.38	0.757	0.514^***^	0.759	0.309^***^	0.779 | 0.672	
ICS-08: Physicians usually asks or midwife’s opinion	2.01	0.945	2.54	0.852	0.519^***^	0.885	0.495^***^	0.723 | 0.648	
ICS-09: Physicians anticipate when midwives need their help	2.19	0.815	2.59	0.800	0.518^***^	0.793	0.500^***^		
ICS-10: Important information is always passed on between midwives and physicians	3.48	0.647	3.65	0.636	0.433^***^	0.683	0.240^***^		
ICS-11: Disagreements with physicians are usually clarified	2.31	0.812	2.64	0.743	0.454^***^	0.815	0.402^***^		
ICS-12: Physicians think their work is more important than the work of midwives^1^	1.86	0.900	2.04	0.910	0.519^***^	0.888	(0.196^**^)^3^		
ICS-13: Physicians are willing to discuss their new practices with us	2.12	0.893	2.39	0.818	0.440^***^	0.908	0.302^***^	0.719 | 0.538	
*Equitable communication (EC)*	*1.80*	*0.435*	*2.59*	*0.624*	*0.481* ^***^	*0.563*	*1.405* ^***^		*0.920 | 0.864*
EC-01: Physicians and midwives nurses consider themselves as a team	1.90	0.528	2.87	0.679	0.388^***^	0.679	1.436^***^	0.838 | 0.733	
EC-02: Physicians and midwives nurses encounter at eye level	1.68	0.486	2.51	0.778	0.391^***^	0.739	1.111^***^	0.875 | 0.825	
EC-03: Professionals try to place themselves in the perspective of the other professional group	1.80	0.519	2.38	0.647	0.354^***^	0.671	0.862^***^	0.809 | 0.685	

Scale properties for the reduced ICS and the EC scale. ^1^Inversely poled item. ^2^Pearson correlation. ^3^not significant after Bonferroni correction (adjusted α = 0.003 for *n* = 18 tests). ^4^Standard deviation of differences between PPC and BC. ^5^Standardized response mean of differences between PPC and BC. ^6^Item-total-correlation of the items of the reduced ICS and the EC scale; ^*^p < 0.05, ^**^p < 0.01, ***p < 0.001; PPC = prenatal/postpartum care; BC = birth care.

Convergent and discriminant validity of the supplemented ICS scale were examined by incorporating established assessment scales as well as newly developed items based on the content of the preceding qualitative interviews. To assess midwives’ *job satisfaction*, the corresponding scale from the Copenhagen Psychosocial Questionnaire (COPSOQ; (Kristensen et al., 2005)) was used. Five aspects of *job satisfaction* (career perspective, people you work with, physical job conditions, organization of work situation, opportunities to contribute skills) are rated on 4-point Likert scales (“1” – “very satisfied” to “5” – “very dissatisfied”). The aggregated scale score proved to be sufficiently internal consistent [Cronbachs α = 0.78; (Nübling et al., 2006)].

In the preceding qualitative interviews *perceptions and attitudes toward the obstetric care process and professional responsibilities* could be identified as relevant for IC between physicians and midwives. To record these in a standardized way, corresponding items were developed. Eleven aspects of *attribution of professional responsibilities in consulting and support* (see Table 2) were answered on 5-point bipolar rating scales. The response categories were chosen to indicate whether the physician or the midwife was considered more responsible (“−2” = “physician”, “−1” = “rather the physician”, “0” = “both equally”, “+1” = “rather the midwife”, “+2” = “midwife”). Eleven items on *attitudes towards obstetric care* (see Table 2) were answered on 6-point bipolar rating scales (“1” – “does not apply at all” to “4” – “applies completely”). Finally, the frequency of collaboration with (1) pediatricians, (2) gynecologists and (3) other midwives and maternity nurses was surveyed by selecting from the categories “never”, “occasionally” and “frequently”.

**Table 2:** Correlation of the reduced ICS and the EC scale with satisfaction with work, responsibility consulting/support, attitudes toward obstetric care as well as frequency of collaboration with other professional groups.

	PPC		BC	
	ICS-R	EC	ICS-R	EC
**Perceptions and attitudes toward the obstetric care process and professional responsibilities**				
*Attitudes toward obstetric care*				
A01 - A clinical birth is usually preferable to a home birth	0.420^***,a^	0.413^***,a^	0.343^***,a^	0.385^***,a^
A02 - Joint supervision of all professional groups involved is essential for good quality in obstetric care	−0.085	−0.038	−0.059	0.127^*^
A03 - Midwives should work more in midwife-led birth centers.	−0.242^***,a^	−0.203^***,a^	−0.300^***,a^	−0.123^*^
A04 - If I know that the physicians have already performed an examination, I prefer to perform it again myself	−0.086	−0.158^**^	−0.145^*^	−0.074
A05 - I think the communication path between the physicians and the midwives *via* the maternity passport/preventive care booklet is sufficient	0.291^***,a^	0.277^***,a^	0.264^***,a^	0.214^***,a^
A06 - Midwives should be allowed to take on more diagnostic tasks (e.g., ultrasound) in the care process	−0.121^*^	−0.139^*^	−0.109	−0.093
A07 - Current financing in obstetrics creates competition between midwives and physicians	−0.325^***,a^	−0.320^***,a^	−0.348^***,a^	−0.223^***,a^
A08 - Midwives are the first point of contact for parents in case of uncertainty, providing referrals to other professionals or facilities	−0.018	0.028	−0.089	−0.001
A09 - Integration of midwifery care in general practices is an important step in ensuring quality of care	0.216^***,a^	0.207^***,a^	0.335^***,a^	0.328^***,a^
*Responsibility consulting/support*				
R01 - Parturient with gestational diabetes	−0.063	−0.083	−0.131^*^	−0.008
R02 - Physiological birth	−0.170^**^	−0.103	−0.181^**^	0.031
R03 - Information about possible complications during birth	−0.215^***,a^	−0.273^***,a^	−0.277^***,a^	−0.225^***,a^
R04 - Breastfeeding counseling	0.025	0.061	−0.061	0.139^*^
R05 - Counseling for pregnant women’s fears and anxieties about childbirth	−0.052	−0.066	−0.091	−0.040
R06 - Treatment of mastitis	0.033	0.062	−0.114^*^	0.000
R07 - Control of the infant heart actions	−0.093	−0.105	−0.200^***,a^	−0.125^*^
R08 - Information about physical changes during pregnancy	−0.086	−0.101	−0.137^*^	−0.047
R09 - Vaccination counseling	0.020	0.019	−0.036	−0.094
R10 - Postpartum courses	−0.037	0.015	−0.071	0.088
R11 - Nutritional counseling	−0.074	−0.084	−0.158^**^	−0.051
**Frequency collaboration professional groups**				
Pediatricians	0.238^***,a^	0.215^***,a^	0.191^**^	0.131^*^
Gynecologists	0.340^***,a^	0.294^***,a^	0.361^***,a^	0.315^***,a^
Other midwives and maternity nurses	0.120^*^	0.072	0.141^*^	0.161^**^
**COPSOQ – Satisfaction with work (scale)**	0.011	0.051	0.101	0.041
C01 - Career perspectives	0.126^*^	0.120^*^	0.176^**^	0.137^*^
C02 - People you work with	0.164^**^	0.163^**^	0.226^***,a^	0.166^**^
C03 - Physical job conditions	−0.088	−0.027	−0.110	−0.097
C04 - Organization of work situation	0.019	0.008	0.007	−0.008
C05 - Opportunities to contribute skills	0.113	0.076	0.136^*^	0.101
C06 - Salary	0.051	0.043	0.103	0.060

PPC = prenatal/postpartum care, BC = birth care; ^*^p < 0.05, ^**^p < 0.01, ^***^p < 0.001. ^a^Still significant after Bonferroni correction (adjusted α = 0.0005 for *n* = 88 tests).

### 2.3. Data analysis

Before starting the in-depth analysis missing values in the scale items were imputed by the expectation maximization (EM) algorithm implemented in the Software SPSS 26. EM-imputation is generally recommended in case of metric or Likert scale items to avoid biases due to possibly not completely random missing values [MCAR; (Schafer and Graham, 2002; Wirtz, 2004)]. Further analyses were started after reverse coding of negatively worded items.

Using the maximum likelihood method, we performed confirmatory factor analyses (CFA; Little and Kline, 2016) to check which of the assumed structural models (uni-, two-or four-dimensional) allows the best fit of the empirical variance–covariance-matrix. For this purpose, a CFA model was defined in which the data of the two care settings [prenatal/postpartum care (PPC) and birth care (BC)] were analyzed in an integrated manner (design for dependent measurements). The possible dependence of the constructs and the items across the care settings was thus taken into account in the modeling approach.

The appropriateness of the CFA models was assessed by measures of global and local fit (Little and Kline, 2016). The *χ*^2^-value allows to test the significance of deviations of the empirical and model implied information in the variance–covariance matrix. However, this test is overly sensitive to sample size (Schermelleh-Engel et al., 2003). Alternatively, measures of approximate fit allow a more valid testing of the global model fit, as they focus on the empirical relevance of inaccuracies of model predictions. The Root Mean Square Error of Approximation (RMSEA) quantifies the amount of unexplained information in the data set. RMSEA less than 0.05 indicates a good model fit (acceptable fit: RMSEA <0.08), because less than 5% of the empirical information remains unexplained. Incremental fit measures like the Confirmatory Fit Index (CFI) and the Tucker-Lewis Index (TLI) reflect a higher model precision the closer their value is to 1 (good model fit: CFI, TLI > 0.97; acceptable model fit: CFI, TLI > 0.95; Schermelleh-Engel et al., 2003). A value of 1 indicates that the tested model can fully explain all the variance–covariance information in the data set. The Bayesian Information Criterion (BIC) makes it possible to compare models of different complexity, since it takes into account the models ‘degrees of freedom (df). Additional df are rewarded by this information-theoretic measure. If the number of analysis variables remains the same, the model with the lowest BIC value provides the best data fit according to the respective df (Schermelleh-Engel et al., 2003; Little and Kline, 2016).

Additionally, at the local item level it must be ensured that each item is sufficiently closely associated with the factor to which it is assigned: factor loadings >0.632 or indicator reliabilities >0.400 indicate an acceptable item-construct association (Little and Kline, 2016).

For the identified scales Cronbach’s α was determined as a measure of internal consistency. According to Classical Test Theory, α is an estimate of the correlation of the aggregated scale value and the underlying latent true score (Lord and Novick, 2008). α > 0.7 indicates acceptable internal consistency. Values above 0.8 indicate good internal consistency.

Paired t-tests were calculated to analyze differences between care settings of IC at scale level (ICS-R and EC scale) and item level [research question 2; (Tabachnick and Fidell, 2014)]. The stability of the scale items across care settings was tested by calculating Pearson product–moment correlations (Tabachnick and Fidell, 2014). The association of the ICS-R and EC-scale with further (care-) characteristics was determined by calculating Pearson product–moment correlations (research question 3). To account for the problem of multiple testing regarding research question 2 and 3, Bonferroni-corrected significance limits are reported (Tabachnick and Fidell, 2014).

All statistical analyses were performed using the statistic software SPSS 26.0 and MPlus 8.3 (Muthén and Muthén, 2017).

## 3. Results

### 3.1. Sample characteristics and descriptive statistics

325 midwives completed the online questionnaire. Of those, 26 respondents (0.8%) were excluded because of limited data quality (proportion of missing values >10% in scale items). Table 3 shows the distribution of key characteristics in midwifery activity and employment. Mostly, participating midwives work as independent midwives (88.8%) in urban areas (66.2%). On average, midwives have 18.64 years of professional experience (median = 18.00, SD = 11.96).

**Table 3:** Descriptive sample statistics.

	*N* (%) Total: 299
**Scope of activity**	
Prenatal/pregnancy care	213 (71.2%)
Birth	174 (58.2%)
Postpartum	275 (92.0%)
**Employment**	
Independent	265 (88.6%)
Private medical practice	34 (11.4%)
Private midwife practice	76 (25.4%%)
Clinic	143 (47.8%)
Obstetric clinic	76 (25.4%)
Perinatal focus	16 (5.4%)
Perinatal center level 1	60 (20.1%)
Perinatal center level 2	17 (5.7%)
Attending midwife	42 (14.0%)
Other	31 (20.4%)
**Volume of work**	
Full-time	152 (50.8%)
Part-time up to 50%	102 (34.1%)
Part-time at least 50%	28 (9.4%)
**Work location**	
Urban area	198 (66.2%)
Rural area	95 (31.8%)
Professional experience (years) [min, 1., 2., 3., quartile, max]	[1.0, 8.0, 18.0, 29.0, 52.0] M = 18.64; SD = 11.96.

Table 1 shows the mean values for the individual items of the original ICS and the supplemented EC items separately for the assessed care settings prenatal/postpartum (PPC) and birth (BC). For 13 of the 16 items, the assessment of IC quality was significantly higher for birth after correcting for multiple testing. The standardized response mean for the original ICS items proved to be small to medium (SRM = 0.240–0.513). The three items on EC indicated very high differences between settings (SRM = 0.862–1.436). Thus, in the birth setting, IC was higher in all assessed aspects. Furthermore, the single items were significantly correlated between PPC and BC setting within the range of medium to high effect sizes (*r* = 0.311–0.560).

### 3.2. Confirmatory factor analysis of competing structural model definitions

Table 4 shows the results of the CFAs for the assumed integrated model structures of the items of IC at BC and in PPC. The one-dimensional model (1 DIM) did not fit the data information adequately (*χ^2^* (*df* = 447) = 1177.79; *p* < 0.001; RMSEA = 0.075 (90%CI: [0.069; 0.080]); CFI = 0.866). The two-dimensional model (2 DIM; ICS, EC) and the four-dimensional model (4 DIM; *Communication*, *Accommodation*, *Isolation*, *EC*) provided a considerably better model fit. For these two models, a similar global data fit could be determined. The fit indicators RMSEA_2DIM/4DIM_ = 0.051/0.052 and CFI_2DIM/4DIM_ = 0.937/0.939 proved to be acceptable.

**Table 4:** Factor loadings and model fit indices for the tested confirmatory model structures for prenatal/postpartum care and birth care.

	1 DIM	4 DIM^2^	2 DIM	2 DIM-R		
	Standardized item loadings (PPC | BC)				Intercept	r_e_^3^
*Communication (ICS-C)*						
ICS-01	0.594 | 0.615^1^	0.616 | 0.630	0.608 | 0.632	–	–	–
ICS-03	0.707 | 0.756	0.718 | 0.772	0.725 | 0.774	0.727 | 0.776	3.10 | 2.70	0.084
ICS-09	0.524 | 0.639	0.530 | 0.643	0.531 | 0.643	–	–	–
ICS-10	0.294 | 0.306	0.268 | 0.299	0.285 | 0.303	–	–	–
ICS-11	0.568 | 0.616	0.574 | 0.547	0.577 | 0.633	–	–	–
*Accomodation (ICS-A)*						
ICS-02	0.578 | 0.747	0.610 | 0.754	0.602 | 0.753	0.572 | 0.745	3.27 | 2.65	0.090
ICS-04	0.691 | 0.663	0.703 | 0.685	0.697 | 0.673	0.702 | 0.674	3.11 | 2.92	0.223
ICS-05	0.739 | 0.801	0.754 | 0.823	0.748 | 0.816	0.772 | 0.827	3.15 | 2.58	0.280
ICS-06	0.652 | 0.753	0.683 | 0.786	0.670 | 0.776	0.682 | 0.784	3,65 | 2.87	0.079
ICS-07	0.706 | 0.794	0.713 | 0.813	0.714 | 0.805	0.725 | 0.818	3.17 | 2.77	0.184
*Isolation (ICS-I)*						
ICS-08	0.706 | 0.748	0.726 | 0.778	0.712 | 0.756	0.700 | 0.743	2.98 | 2.23	0.326
ICS-12	−0.501 | −0.328	−0.528 | −0.343	−0.501 | −0.318	–	–	–
ICS-13	0.551 | 0.704	0.592 | 0.728	0.561 | 0.705	0.537 | 0.689	2.85 | 2.34	0.191
*Equitable communication (EC)*						
EC-01	0.726 | 0.778	0.814 | 0.875	0.814 | 0.875	0.813 | 0.874	3.49 | 2.34	0.110
EC-02	0.775 | 0.826	0.918 | 0.940	0.916 | 0.941	0.916 | 0.941	2.77 | 2.35	0.046
EC-03	0.637 | 0.750	0.736 | 0.857	0.738 | 0.856	0.741 | 0.858	3.06 | 2.57	0.229
Correlation of the ICS construct between care settings (PPC and BC)	r_1DIM_ = 0.728	*r*_ICS-C_ = 0.635 *r*_ICS-A_ = 0.727 *r*_ICS-I_ = 0.753 *r*_EC_ = 0.578	*r*_ICS_ = 0.705 *r*_EC_ = 0.578	*r*_ICS_ = 0.724 *r*_EC_ = 0.579		
Correlation of the constructs within the care setting PPC	–	*r*_ICS-C, ICS-A_ = 0.973 *r*_ICS-C, ICS-I_ = 0.988 *r*_ICS-A, ICS-I_ = 0.936 *r*_ICS-C, EC_ = 0.774 *r*_ICS-A, EC_ = 0.795 *r*_ICS-I, EC_ = 0.810	*r*_ICS,EC_ = 0.802	*r*_ICS,EC_ = 0.801		
Correlation of the constructs within the care setting BC	–	*r*_ICS-C, ICS-A_ = 0.988 *r*_ICS-C, ICS-I_ = 0.996 *r*_ICS-A, ICS-I_ = 0.927 *r*_ICS-C, EC_ = 0.761 *r*_ICS-A, EC_ = 0.770 *r*_ICS-I, EC_ = 0.775	*r*_ICS,EC_ = 0.779	*r*_ICS,EC_ = 0.774		
*Global fit measures*						
χ	1177.79	751.98	785.73	226.35		
df	447	420	442	192		
p	<0.001	<0.001	<0.001	0.045		
TLI	0.851	0.928	0.929	0.990		
CFI	0.866	0.939	0.937	0.991		
RMSEA [90%CI]	0.075 [0.069; 0.080]	0.052 [0.046; 0.058]	0.051 [0.046; 0.057]	0.025 [0.004; 0.037]		
SRMR	0.055	0.047	0.050	0.032		
AIC	19708.53	19336.72	19326.47	–^4^		
BIC	20124.77	19852.43	19761.13	–^4^		

^1^Factor loadings. ^2^Not positive definite. ^3^Residual correlation PPC, BC. ^4^Not suitable for model comparison due to reduced number of items.

However, the four-dimensional model was not factorial valid due to the exceptionally high correlations of the three subfactors of ICS. *Communication* correlated with *Accommodation* and *Isolation* in the care settings PPC and BC to. 973/0.988 and 0.988/0.996, respectively. The correlation of *Accommodation* and *Isolation* in the care settings PPC and BC was 0.936/0.927. Hence, the separability of these three components proved to be not possible due to the high information redundancy. Overall, the three-factor structure found in the original version of the ICS proved not to be appropriate in the sample of midwives. A second distinct construct, in addition to the ICS component, resulted only from the newly added EC items.

In all models tested, some of the ICS items exhibited insufficient factor loadings and thus insufficient factor reliabilities. In particular item ICS-10 (“*Important information always passed on*”) (max. loading = 0.303) falled substantially below the critical threshold of 0.642. The loadings of items ICS-01 (“*Good understanding with physicians about our respective responsibilities*”), ICS-09 (“*Anticipate when midwives need their help*”), ICS-11 (“*Disagreements with physicians are often resolved*”), and ICS-12 (“*Consider their work more important*”) were below 0.624. After removing these items from the model, the reduced model definition 2-DIM-R yielded an excellent global model fit: *χ*^2^_(df = 192)_ = 226.35, *p* = 0.045; RMSEA = 0.025 (90%CI: [0.004; 0.037]); CFI = 0.991. At the local fit level, especially for the BC setting, the item-construct associations proved to be good (min. loading: 0.674). The item-specific residual correlations across the two care settings, as indicators of the information stability that cannot be explained by the latent constructs, are low or at most moderate (*r_e_* = 0.046–0.326; Table 4). This substantiates the adequacy of the assumed structural model, as setting-relevant information was adequately represented by the ICS-R and EC constructs (stability across care settings: *r*_ICS-R_ = 0.724, *r*_EC_ = 0.579).

Within the care settings the intercorrelation of ICS-R and EC was 0.801 and 0.774, respectively.

### 3.3. Scale properties of the ICS-R and EC scales regarding care settings

**Figure 1:**
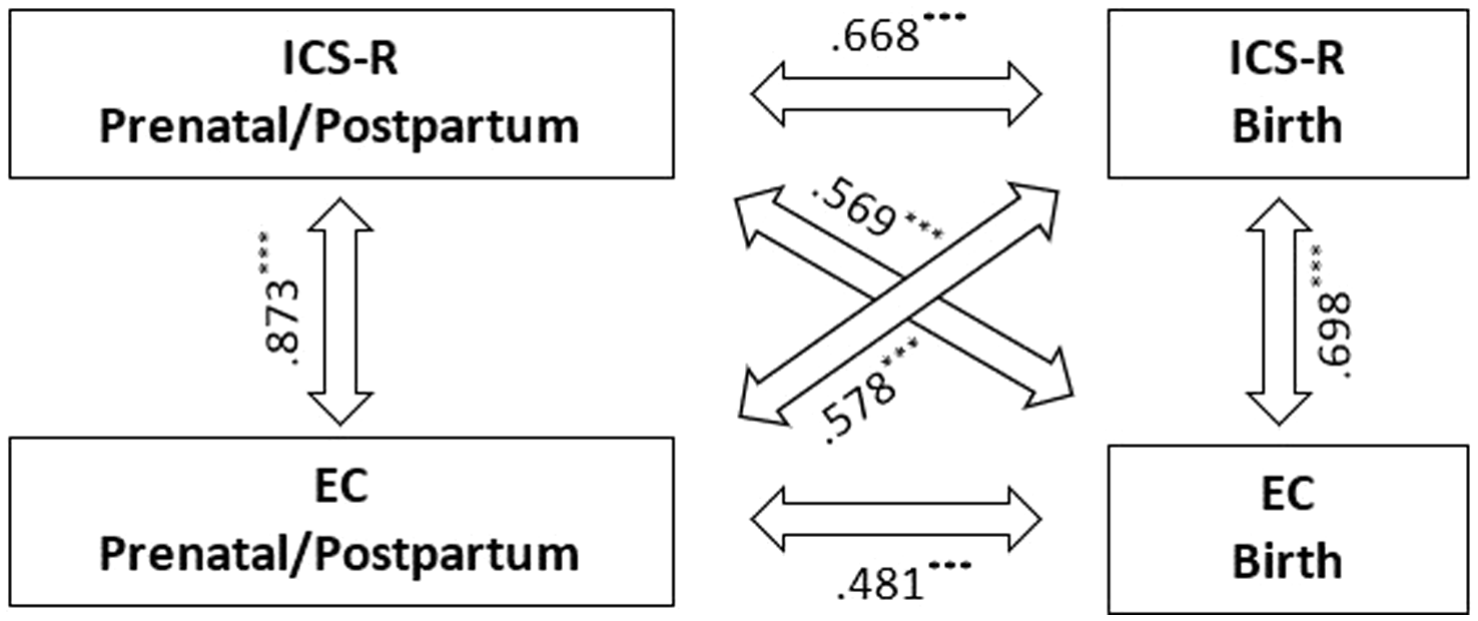
Correlation of the reduced ICS and the EC scale for both prenatal/postpartum and birth care (^***^p < 0.001).

Sufficient corrected item-total correlations and internal consistencies were obtained for both scales (Table 1). For PPC scores were slightly higher (*r*_it_ = 0.651–0.799 and 0.809–0.875, respectively; *α* = 0.920/0.920, respectively) than for BC (*r*_it_ = 0.546–0.695 and 0.685–0.825, respectively; *α* = 0.874/0.864). The ICS-R-and EC-scale proved to be highly correlated within in both settings: *r*_PPC_ = 0.873, *r*_BC_ = 0.698 (Figure 1). Stability between the two care settings was more pronounced for the ICS-R scale (*r* = 0.668) than for the EC scale (*r* = 0.481). These scale intercorrelations thus correspond very well with those at the latent construct level (Table 4).

Both scales reflected a higher degree of IC between midwives and physicians in BC than in PPC (see Table 1). The ICS-R scale showed a medium effect size of SRM = 0.579 between the two care settings. The fact that the difference on the EC scale was even more pronounced with SRM = 1.405 is due to the considerably lower scale mean in PPC (*M*_PPC_ = 1.80 vs. *M*_BC_ = 2.25).

### 3.4. Correlation of the final scales (2-DIM-R) with other work characteristics and assessments of the midwives

When midwives are more likely to prefer clinical birth (A01, *r* = 0.343–0.420) and when they are more likely to communicate with physicians indirectly (maternity passport, A05, *r* = 0.214–0.291), satisfaction with IC tends to be higher on both scales in both settings (Table 2). This is also consistent with midwives who are more satisfied with IC having a desire to integrate midwifery care into general practices (A09, *r* = 0.207–0.335) and being more critical of midwife-led birth centers (A03, *r* = −0.203 to −0.300). The more frequently midwives work together especially with gynecologists (*r*_ICS-R_ = 0.340/0.361; *r*_EC_ = 0.294/0.315) but also with paediatricians in PPC, the more positive is their view on IC as well as EC (*r*_ICS-R_ = 0.238; *r*_EC_ = 0.215). The *COPSOQ - Satisfaction with work scale* was not correlated with both scales in both settings (Table 2). One exception was item C02: the more satisfied midwives are with their cooperation with other people during birth, the higher they rate the ICS-R (*r* = 0.226). Also with regard to the area of *Responsibility Consulting/Support*, only one item (R03) showed a significant correlation after Bonferroni correction in both settings (*r* = −0.215 to −0.277). When midwives see themselves as primarily responsible for passing on information about complications during birth, they are less satisfied with IC on both scales.

## 4. Discussion

In this study, the ICS was used to assess IC between midwives and physicians for the first time (Kenaszchuk et al., 2010; Vittadello et al., 2018). The ICS was expanded to include Equitable Communication (EC) in order to validly represent IC in midwifery and obstetric care. Our results suggest that the adapted ICS-R/EC assessment allows to capture perceived IC in a psychometrically sound manner. The setting-specific operationalization supports the recommendation of Vittadello et al. (2018) that different “allied health professional” disciplines and their action settings should be considered separately. This takes into account that IC between physicians and individual subgroups of allied health professionals may take different forms and qualities based on the particular profession-specific concept of care and intensity in terms of patient contacts.

### 4.1. Research question 1: structural properties of the ICS from the midwives’ perspective

The a priori tested three-dimensional structure of the original ICS (Kenaszchuk et al., 2010; Vittadello et al., 2018) could not be confirmed in the sample of midwives. In both examined care settings PPC and BC the two-dimensional structure proved to be superior, after considering insufficient item-construct association of 5 ICS items. In contrast to existing research findings on IC between physicians and nursing, midwives seem to perceive the ICS-facets *Communication*, *Accommodation*, and *Isolation* less differentiated and more in terms of general IC. However, it must also be taken into account that in previous studies using the ICS in primary health care (cooperation physicians, nursing, allied health personnel), the theoretically postulated subconstructs proved to be poorly separable: E.g., high scale intercorrelations of the *Communication* facet with the *Isolation* and *Accommodation* facets (*|r|* = 0.78–0.86) were found (Kenaszchuk et al., 2010). Accordingly, the Fornell-Larcker discriminant factorial validity criterion proved to be violated, because the item-construct associations fell below the according scale intercorrelations substantially (Fornell and Larcker, 1981). In addition, a confirmatory test of model fit differentiated by rater-target group combinations (nurse vs. physician; allied professional vs. physician; physician vs. nurse; allied professional vs. nurse; nurse vs. allied professional; physician vs. allied professional) also indicated an insufficient to weak model fit for the assumed three-dimensional structure (CFI = 0.823–0.948; TLI = 0.904–976) (Kenaszchuk et al., 2010).

Due to insufficient indicator reliabilities, 5 ICS items were eliminated. Item ICS-12 (“*Consider their work is more important than ours*”) represents the only negatively worded item in the entire ICS, which may contribute to the poor item fit in the overall model. The remaining 4 eliminated ICS items (ICS-01, ICS-09, ICS-10, ICS-11) indicate the original ICS subfacet *Communication* according to Kenaszchuk et al. (2010). The study of Vittadello et al. (2018) also showed a considerably weaker loading for the 10th item (“*Important information is always passed on from us to the other professio*n”) in the German translated version than in the original English version (Kenaszchuk et al., 2010). The insufficient item-construct association of this item may be caused from a semantic shift occurred during the translation process of the German version by Vittadello et al. (2018). In the German version respondents rate the extent to which their own profession transmits information to the other profession (“*Important information is always passed on* ***from*** *us* ***to*** *the other profession*” [ICS-10 German translation] (Vittadello et al., 2018)). In the original version the responsibility for the transmission of information is not attributed to one of the interacting professional groups: “*Important information is always passed on* ***between*** *us* ***and*** *them*” (ICS-10 original). In general, the ICS is designed to evaluate primarily the behavior of the respective other professional group with regard to IC with one’s own professional group (external evaluation). The focus is less on the assessment of the extent to which one’s own professional group practices interprofessional behavior (self-assessment). This minimal linguistic shift may lead to (i) weaker indicator reliability and (ii) a bias due to socially desirable response behavior, self-serving bias, being more pronounced in the German version than in the original form (Dufner et al., 2018).

Item ICS-09 (“*Anticipate when midwives need their help*”) addresses less strongly active verbal communication behaviors. This item relates more to aspects of work organization in terms of supportive collective action or the concept of *Collective Intelligence* (Jean et al., 2020). *Collective Intelligence* is positively related to IC in healthcare but should be considered as an independent information component (Awal and Bharadwaj, 2014). In contrast, Item ICS-11 (“*disagreements with physicians are often resolved*”) primarily addresses the conflict culture within the team to overcome the described professional dissonance in midwife-physician teams, rather than specific communication skills (McFarland et al., 2020). Item ICS-01 (“*Good understanding with physicians about our respective responsibilities*”) deals with the aspect of perspective adoption. The adaptation of the perspectives and concepts of other reference disciplines as well as an active reflection of one’s own actions characterizes the highest level of collaboration (transdisciplinary). This allows the creation of a common understanding, which would not have been possible without the formation of synergies (WHO, 2010).

Due to the item selection, the aspect of *Communication* is thus significantly weaker represented in the ICS-R compared to the original ICS. Instead, the aspect of *Equitable Communication* (EC) has proven to be a clearly separable alternative communication facet. EC addresses in particular interactional factors, i.e., communication behavior that promotes group esteem and internal cohesion (D’Amour et al., 2008; Behruzi et al., 2017). Perceived boundaries or inequalities among members in an interprofessionally designed care team represent a key barrier to the implementation of IC in practice (Aquino et al., 2016). Interpersonal appreciation within a team represents a facilitating factor, as it implies the individual’s need for recognition, consideration, and acceptance (Warschburger, 2009; Behruzi et al., 2017). Thus, conflictual IC processes in the obstetric setting have been attributed partly to the lack of appreciation (Behruzi et al., 2017).

In summary, the ICS-R/EC-assessment allows for a comprehensive and psychometric sound examination of the IC domain in woman-centered midwifery and obstetric care.

### 4.2. Research question 2: differences between care settings from the midwives’ perspective

Overall, midwives rated IC and EC with physicians in PPC as rather unsatisfactory (*M*_PPC_ = 1.68–2.40). Considerably better values are obtained for BC (*M*_BC_ = 2.38–2.87). This is in line with existing findings from previous studies that perceived IC with physicians is rated as more critical by midwives (Warmelink et al., 2017; Romijn et al., 2018).

An analysis at the individual item level reveals that the differences are reflected to different degrees (item-stetting interaction). While weak to moderate differences appeared for the 8 ICS items, large effects were found for the EC items (SRM = 0.862–1.436). The overall EC within the midwife-physician care dyad turned out to be more pronounced in BC than in PPC (SRM = 1.405) (WHO, 2010).

Furthermore, discrepancies between care settings may result from specific characteristics of the health care system in general and the according model of care (Scheerhagen et al., 2015). While in BC care is usually provided by an interprofessional team at one location, PPC is organized multiprofessionally, autonomously in the sense of parallel care (Careau et al., 2015). Because the fields of action and communication situations are separated in the latter setting, less direct coordination is feasible and necessary, so that perspectives and concepts of the reference disciplines may be reflected to a lesser extent (WHO, 2010).

Furthermore, the results provide evidence that IC is mainly judged as satisfactory when midwives have similar birth and care related concepts and attitudes as physicians (O’Reilly et al., 2017). This is characterized by a more clinically oriented view, preferring clinical births to home births, accepting light forms of IC (information and communication via the maternity passport), and considering collaboration with physicians in private practice. Satisfactory IC may be supported if midwives work primarily in the clinical setting and experience a socialization process similar to that of the medical profession (O’Reilly et al., 2017).

### 4.3. Research question 3: association of the ICS with further care and IC characteristics

In contrast to existing study results, no or only weak correlations between the IC with the COPSOQ-items (Kristensen et al., 2005) on job satisfaction could be identified (Hansson et al., 2022). It should be regarded that existing studies on midwives’ job satisfaction analyze only IC sub-facets [e.g., role conflict (Stahl et al., 2019); lack of appreciation (Weller et al., 2014); recognition (Papoutsis et al., 2014)]. Primarily workload, salary, work-life balance, and autonomy proved to be significantly associated with midwifes’ job satisfaction and early career exits (Kirkham et al., 2006; Jarosova et al., 2016; Nedvědová et al., 2017; Hansson et al., 2022). The quality of IC should thus be assumed to be primarily a moderator rather than a central predictor of job satisfaction in midwifery care.

In addition to a good organizational structure and sufficient available resources, experience with IC represents an important determinant of successful IC (Downe et al., 2010). The present results confirm these findings. The higher the frequency of collaboration with other professional groups, the better the overall assessment of IC and EC in all care settings studied (*r* = 0.215 to 0.361). The associations with IC with pediatricians proved to be weaker compared to IC with gynecologists. This is reasonable because of the job-related responsibilities, especially in the obstetric setting (pediatricians are not involved in obstetrics). This is in line with the call to establish IC processes early in the respective training programs of all disciplines involved (e.g., midwives, physicians, nursing) (Stahl and Agricola, 2021).

External framework conditions and professional positions determine which responsibilities for midwifes and physicians exist, and which instance is accountable for them (Auhagen, 2002). In Germany, this is not always clearly defined, especially due to legal regulations. For example, insured women with no risk are allowed to receive services during pregnancy (exception: sonography) from a physician, a midwife, or both (§134a social code V). Furthermore, women without abnormal (pathological) progress are able to choose between a clinical or a non-clinical (home birth, birth center, midwife’s office) delivery. Accordingly, some areas of responsibility cannot be clearly assigned to a single professional group. Thus, emerging role conflicts or unclear areas of responsibility represent a central challenge for the implementation of successful IC in German midwifery care (Aquino et al., 2016; Stahl, 2016). The results indicate that IC is rated as satisfactory especially when midwives tend to assign responsibility to physicians in highly midwifery-specific areas of activity related to direct birth care (e.g., information about possible complications during birth, control of infant heart action; Table 2). This is in line with existing evidence, suggesting a need for action to reduce role conflict between midwives and physicians in order to improve existing IC processes (Hansson et al., 2020).

### 4.4. Limitations

In this study, self-rating data were analyzed, which reduces the validity due to methodological limitations. Subjective judgments may be specifically influenced by response sets (e.g., self-serving bias, social desirability, consistency effects, halo effects due to positive care experiences during birth) (Dufner et al., 2018). Furthermore, the ad-hoc study sample may distort the distribution of relevant midwifery-specific characteristics in health care practice (e.g., skewed urban/rural ratio) (Higgins et al., 2020). Because the present study was designed as a cross-sectional survey to collect retrospective judgments, considerations about possible causal effects have only limited empirical evidence and should be interpreted with caution (Dufner et al., 2018). The accuracy and validity of the judgment depends not only on the competence of the participating midwives and the quality of the IC, but also by the extent of experience that could be acquired in the IC with the other profession (Neyer, 2006). Due to fewer communication needs and opportunities within the multiprofessional collaboration in PPC setting, external judgments may therefore be biased to a greater extent, e.g., by tendency to extreme values or halo-effects (Dufner et al., 2018). To reduce potential individual judgment biases, there is a need for greater aggregation of external judgments of midwives who work predominantly in the PPC setting.

### 4.5. Research perspectives and conclusion

In general, further validation steps of the ICS-R and EC scale seem necessary. In addition to the data for midwives, the physicians’ perspective should be analyzed in an integrated way (Neyer, 2006). Simultaneously analyzing and comparing the perspectives of both professional groups is an essential prerequisite for obtaining a more complete view on everyday care-related IC processes. Assessing the perspective of physicians (gynecology, obstetrics, and pediatrics) is important since they significantly regulate the involvement of others in teamwork and take responsibility with regard to an effective allocation of work resources (O’Reilly et al., 2017). The comparative and integrated consideration of different perspectives of professions involved in care creates the basis for being able to differentiate coordination behavior as well as interaction patterns in the team. This may help to identify in which environment and in which conditions IC processes can be established appropriately. Particularly, future surveys should examine (i) whether the two-dimensional structure of the assessment instrument is also valid in the physicians’ population, and (ii) to what extent the identified setting-specific differences (prenatal, perinatal, postpartum) represent a specific feature of midwifery work or rather a generally valid feature in the interprofessional care of (expectant) mothers. Regarding the construct IC in general, it should be investigated which aspects of the IC construct can be considered generic and overarching and with regard to which aspects adjustments are necessary depending on the investigated collaborating professional dyads. Adopting analysis procedures based on generalizability theory (Brennan, 2001) provides the opportunity to systematically differentiate overlapping information components of assessment data in order to (i) identify their importance for midwives’ and physicians’ IC assessment and (ii) understand which information components should be considered for an appropriate interpretation of IC assessment data in future surveys (e.g., assessment perspective, setting, item content) (Brennan, 2001).

The present study expands the focus of IC to include a broader network of health professionals in maternal and neonatal health care. Professions contribute different skills and knowledge to care with the goal of providing the best possible patient care and safety (O’Reilly et al., 2017). The findings provide evidence to improve IC. Early experience of IC processes seems useful to (i) increase the frequency of collaboration, (ii) establish similar socialization processes on an early stage, and (iii) avoid potential conflict in the long term due to varying attitudes towards obstetric care and responsibility in consulting (Romijn et al., 2018). Especially in PPC, the development of appreciative communication and internal team cohesion seems to be particularly challenging. The psychometrically tested two-dimensional ICS-R/EC-instrument provides a validated assessment basis to analyze IC practice in the complex everyday care of midwifery and obstetric care from multiple perspectives, to characterize IC processes between midwives and physicians, and to identify challenges (practice gaps). Understanding how physicians and midwives conceive IC and how it is implemented in daily care is a key prerequisite for identifying problems, exploring approaches to optimize IC processes, implementing them in evaluation processes, and examining the overall effects of successful IC on woman-centered care outcomes (O’Reilly et al., 2017).

## Data availability statement

The raw data supporting the conclusions of this article will be made available by the authors upon request (anja.schulz@ph-freiburg.de), without undue reservation.

## Ethics statement

The studies involving human participants were reviewed and approved by Ethics Committee of the German Psychological Society (Ref: MAW 022019). The patients/participants provided their written informed consent to participate in this study.

## Author contributions

AAS and MAW planned and conducted the data collection, analyzed and interpreted the data set using confirmatory factor analysis, and were primarily involved in all steps of the study and in editing the manuscript. All authors contributed to the article and approved the submitted version.

## Funding

The research project took place within the framework of the project “Analysis of midwifery care in rural areas,” which was initiated by the Network for Families and Midwives Ortenaukreis. The project was supported by the German Federal Ministry of Agriculture and Food from 2017 to 2019 (grant number: 2817LEO15). This publication was funded by the German Research Foundation (DFG) grant “Open Access Publication Funding / 2023-2025 / University of Education Freiburg (512888488).

## Conflict of interest

The authors declare that the research was conducted in the absence of any commercial or financial relationships that could be construed as a potential conflict of interest.

## Publisher’s note

All claims expressed in this article are solely those of the authors and do not necessarily represent those of their affiliated organizations, or those of the publisher, the editors and the reviewers. Any product that may be evaluated in this article, or claim that may be made by its manufacturer, is not guaranteed or endorsed by the publisher.
